# Effect of Pre-Hospital Intravenous Fluids on Initial Metabolic Acid-Base Status in Trauma Patients: A Retrospective Cohort Study

**DOI:** 10.3390/metabo13080937

**Published:** 2023-08-10

**Authors:** Damien Bossel, Mylène Bourgeat, Olivier Pantet, Tobias Zingg

**Affiliations:** 1 Faculty of Biology and Medicine, Lausanne University, 1011 Lausanne, Switzerland; 2 Department of Visceral Surgery, Centre Hospitalier Universitaire Vaudois—CHUV, Lausanne University Hospital, 1011 Lausanne, Switzerland; 3 Service of Adult Intensive Care Medicine, Centre Hospitalier Universitaire Vaudois—CHUV, Lausanne University Hospital, 1011 Lausanne, Switzerland

**Keywords:** balanced solutions, normal saline, pre-hospital, trauma, hyperchloremia, fluid resuscitation

## Abstract

Despite its known harmful effects, normal saline is still commonly used in the treatment of hypovolemia in polytrauma patients. Given the lack of pre-hospital research on this topic, the current study aims to assess the current practice of fluid administration during the pre-hospital phase of care and its effects on initial metabolic acid-base status in trauma patients. We extracted and completed data from patients recorded in the Lausanne University Hospital (CHUV) trauma registry between 2008 and 2019. Patients were selected according to their age, the availability of a blood gas analysis after arrival at the emergency room, data availability in the trauma registry, and the modality of arrival in the ED. The dominantly administered pre-hospital fluid was normal saline. No association between the type of fluid administered during the pre-hospital phase and the presence of hyperchloremic acidosis in the ED was observed.

## 1. Introduction

Massive bleeding is one of the first avoidable causes of death in polytrauma patients [1]. Its treatment aims to stop the bleeding as quickly as possible. To maximize perfusion and tissue oxygenation until hemostatic intervention can occur, the lost intravascular volume is replaced, starting in the pre-hospital setting by the administration of intravenous fluids (IVF) [2].

In the Swiss pre-hospital field, the two most commonly used IVFs are NaCl 0.9%, also called normal saline (NS) solution, and Lactated Ringer’s (LR) solution, which is considered a balanced solution (BS) because of its electrolyte contents, which are similar to those in human plasma.

Due to its supraphysiological chloride concentration and the role of the latter in acid-base homeostasis [3], the administration of large volumes of NS may cause hyperchloremic metabolic acidosis [4,5,6]. This solute also tends to produce hyperkalemia and renal ischemia, as well as vasodilatation, which can worsen the patient’s hemodynamic state [5,7,8,9,10]. Just as acidosis is known to be harmful, hyperchloremia, per se, seems to be associated with increased mortality in major trauma patients [10,11,12,13,14,15]. In addition to its diverse deleterious side effects, hyperchloremic acidosis induced by NS may lead clinicians to an erroneous interpretation of the initial arterial blood gas (ABG), and therefore lead to inappropriate fluid loading [16].

Despite its known harmful effects, NS is still commonly used in the treatment of hypovolemia in polytrauma patients in the field. To this day, no study has investigated the link between pre-hospital NS administration and hyperchloremic acidosis on hospital admission, as well as the outcome of patients in the short and mean term.

The primary aim of our study was to assess the past and current prevalence of pre-hospital NS administration and hyperchloremic acidosis among polytrauma patients during their initial management in the emergency department. The secondary aims were to analyze the potential impact of hyperchloremic acidosis on subsequent management (intrahospital fluid volume administration, intensive care unit (ICU) and hospital length of stay (LOS), and the duration of mechanical ventilation), and the mortality of major trauma patients.

## 2. Materials and Methods

This is a retrospective cohort study based on the registry of major trauma patients at the Centre Hospitalier Universitaire Vaudois (CHUV, Lausanne, Switzerland). Since this is a quality control study, approval from the ethics committee was not required.

### 2.1. Patients

The study population includes all patients recorded in the CHUV trauma registry between 2008 and 2019, a total of 3700 patients. Study inclusion criteria were trauma patients with an age older or equal to 16 years with an ABG available within 12 min after arrival in ED. This limit was chosen because it was the median time interval between arrival and ABG for the patient cohort before the application of all exclusion criteria.

**Figure 1:**
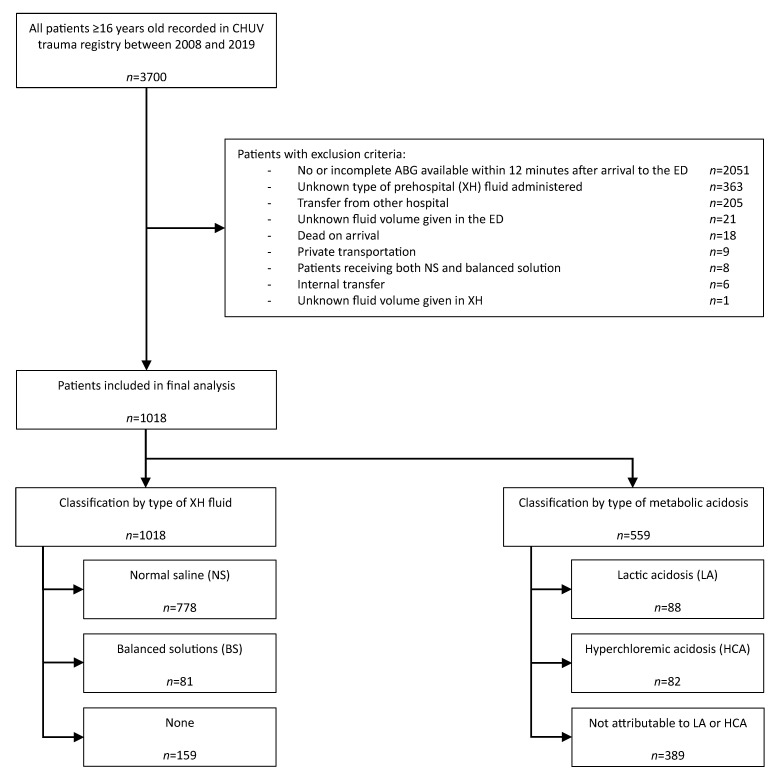
Study population.

In total, 2682 patients were excluded for the following reasons: no or incomplete ABG available within 12 min after arrival to the ED (*n* = 2051), unknown type of pre-hospital (XH) fluid administered (*n* = 363), transfers from other hospitals (*n* = 205), unknown fluid volume given in the ED (*n* = 21), patients dead on arrival (*n* = 18), arrival with private transportation (*n* = 9), patients receiving both NS and balanced solution (*n* = 8), internal transfers (*n* = 6) and one patient with an unknown fluid volume given in XH (*n* = 1). Finally, 1018 patients were included for analysis in our study (Figure 1).

### 2.2. Data Collection

For each patient included in the study, the type and quantity of fluid perfused in the pre-hospital phase (NS versus BS), as well as the intrahospital fluid volumes administered, including packed red blood cell (PRBC) transfusions during the first 24 h, were recorded. The type (arterial vs. venous) and results of ABGs, including pH, base excess (BE), levels of plasma lactate, chloride, sodium, and creatinine, as well as the strong ion difference (SID) at different timepoints during the hospital stay (ER, OR and ICU) were extracted to follow the evolution and determine the origin of any metabolic acid-base derangement. We also reported the necessity of pre-hospital intubation, as well as Glasgow Coma Scale (GCS), heart rate (HR), systolic blood pressure (SBP) and vasopressor use on arrival in the ED. General information like age, gender, mechanism of injury, ISS, mode of transportation, 48-h and 30-day mortalities were also recorded. For the time of hospitalization, we reported the peak creatinine and chloride levels; the presence of acute kidney injury (AKI); time to normalize the BE and clear lactate levels; the need for, and duration of, mechanical ventilation; the need for treatment in the intensive care unit (ICU) or the operating room (OR); and the lengths of stay in the ED, ICU and hospital.

Metabolic acidosis was diagnosed when the BE was <−2 mmol/L in the presence of a pH < 7.45 and the absence of a primary respiratory alkalosis or acidosis. The rationale behind this definition is the fact that metabolic acidosis may be present with a normal pH (7.35–7.45) because of the physiological respiratory compensation through an increase in minute ventilation, resulting in a decreased pCO_2_. When available, the type of metabolic acidosis was determined according to the main effector on the BE (hyperchloremia versus lactate). Anions other than chloride and lactate (mainly albumin, unavailable for most cases) were ignored for the purpose of this study. The effect of lactate was defined as the lactate level in mmol/L. The negative deviation in mmol/l from the normal SID (Plasma Na—Plasma Cl; N: 35 mmol/L) was chosen as the surrogate for the chloride effect on the BE [17]. When the main effector on the BE was an abnormal SID, the origin of the metabolic acidosis was considered as hyperchloremic. Normal values for BE were −2 to 2 mmol/L and lactate levels < 2.44 mmol/L were considered as normal, as defined by our central laboratory.

AKI was defined as the presence of a peak creatinine level 1.5 times above baseline during the hospital stay. Normal creatinine levels were considered as 62–106 μmol/L for men and 44–80 μmol/L for women.

Time to normalization of BE or lactate levels was defined as the time from the initial abnormal value to the first measured normal value (BE > −2, lactate < 2.44).

### 2.3. Outcomes

The primary outcomes were the prevalence of pre-hospital administration of NS and the incidence of hyperchloremic acidosis in the ER.

Secondary outcomes were 48-h and 30-day mortalities, AKI, hospital and ICU lengths of stay, the need for intubation and the duration of mechanical ventilation, and total intrahospital fluid volume including PRBC transfusion.

### 2.4. Statistics

Statistical analyses were performed using Stata Statistical Software: Release 15 (StataCorp. 2017, College Station, TX, USA). For qualitative variables, results are expressed in frequencies and percentages. For continuous variables, a measure of dispersion was given using mean with standard deviation (SD), and median, with lower and upper interquartile ranges (IQR) if the distribution of data was skewed. Qualitative variables were compared using Fisher’s exact or χ^2^ test. Continuous variables were compared using the Student’s *t*-test when distribution was bell-shaped and using a Kruskal–Wallis or Mann–Whitney U-test if distribution was skewed. Differences were considered statistically significant for *p*-values < 0.05.

## 3. Results

A total of 1018 patients were enrolled in our study. The mean age was 44.4 (SD 20) years, and 770 (76%) patients were men.

### 3.1. Results by Type of Pre-Hospital Fluid

Table 1 summarizes the patient characteristics of the study cohort, according to the type of fluid administered during the pre-hospital phase.

In total, 778 (76%) patients received NS, 81 (8%) received BS and 159 (16%) received no fluid. The majority of NS was administered during the time period 2008–2013, with 472 (61%) cases compared to 31 (38%) for BS. The 2014–2019 period was the one in which the majority of the patients received BS, with 50 (62%) cases and 306 (39%) cases for NS (*p* < 0.001).

#### 3.1.1. Mode of Transport and Pre-Hospital Interventions

Ninety-four percent of patients (*n* = 76) who received BS and 50% of patients (*n* = 389) who received NS were brought in by helicopter (*p* < 0.001).

We observed that 22 (27%) BS patients were intubated pre-hospital, which is slightly, but statistically, higher than the 139 (18%) NS patients (*p* = 0.041). Patients in the BS group also received significantly more fluid volume in the pre-hospital setting, with a mean of 763 (SD 607) ml compared with 520 (SD 387) ml for patients in the NS group (*p* < 0.001).

#### 3.1.2. Initial Condition on Arrival in the ED

There was no difference in the ISS between the three groups, which was 16 (SD 12), 19 (SD 14) and 14 (SD 11), respectively (*p* = 0.41). In the ED, for BS and NS patients, the mean GCS was 11 (SD 5.2) and 12 (SD 4.8) (*p* = 0.01). The mean SBP was 133 (SD 28) mmHg and 135 (SD 26) mmHg (*p* = 0.97), the mean HR was 92 (SD 23) beats per minute (bpm) and 90 (SD 22) bpm (*p* = 0.51).

There was no significant difference in the ABG on arrival in ED. For patients in the BS group, 23 (28%) had a normal ABG, 48 (59%) had metabolic acidosis and 10 (12%) had another acid-base derangement. For those in the NS group, 197 (25%) had a normal ABG, 425 (55%) had metabolic acidosis and 156 (20%) had another acid-base derangement (*p* = 0.30).

Median ED length of stay was 55 min (IQR 47) and was significantly longer for the BS group, with 63 min (IQR 63) compared to 55 min (IQR 43) for the NS group (*p* < 0.001).

#### 3.1.3. Evolution and In-Hospital Interventions

The mean intrahospital fluid volume was 2221 mL and there was no significant difference between the two groups (*p* = 0.08). Patients in the BS group received more PRBC than those in the NS group, with a mean of 1.35 compared to 0.53 PRBC during the first 24 h, respectively (*p* = 0.005). Nonetheless, there was no difference in vasopressor need, which was used in 25 (31%) patients in the BS group and 166 (21%) patients in the NS group (*p* = 0.06).

Patients who received BS went more frequently to the OR, with 39 (48%) cases and 236 (30%) cases for those who received NS (*p* = 0.001). ICU hospitalization was necessary in 35 (43%) cases for BS patients and in 272 (35%) for NS patients, which is not statistically different (*p* = 0.26).

The BS group required invasive ventilation in 30 (37%) cases, with a median length of 3.5 (IQR 7) days; in the NS group it was, respectively, 240 (31%) cases (*p* = 0.33) for a median duration of 2 (IQR 7) days (*p* = 0.87).

Fourteen (18%) patients who received BS and 180 (26%) patients who received NS had abnormal creatinine levels (*p* = 0.25), with a creatinine peak of 95 (SD 45) µmol/L and 97 (SD 42) µmol/L (*p* = 0.81), and an AKI incidence during hospitalization in 7 (10%) and 34 (5%) patients, respectively (*p* = 0.07). Comparing BS and NS groups, the median time to normalize BE was 14.7 h (IQR 15.4) and 11.9 h (IQR 19) (*p* = 0.53), and the time to clear lactate was 4.4 h (IQR 11.8) and 5.2 h (IQR 8.6), respectively (*p* = 0.37).

ICU length of stay was longer for BS patients, with 3.5 days compared to 2.3 days for NS patients (*p* = 0.006). However, the median total length of hospital stay was 7 days (IQR 15.5) for the BS group and 5.8 days (IQR 15.5) for the NS group, which is not significant (*p* = 0.15).

#### 3.1.4. Mortality

There was no difference in mortality between the three groups. For BS, NS and “No fluids” groups, 48-h mortality was 9%, 7% and 5%, respectively (*p* = 0.53). The 30-day mortality was 12%, 11% and 10%, respectively (*p* = 0.86).

### 3.2. Results by Type of Metabolic Acidosis

Table 2 summarizes the findings comparing the patients with hyperchloremic (HCA) and lactic acidosis (LA).

Among all patients, 559 (55%) had metabolic acidosis. Table 3 summarizes the main findings by acidosis severity in this group. Patients who had not received any pre-hospital fluids significantly more often had an acidosis with a normal pH. For 170 patients, the necessary data were available to attribute the acidosis to a hyperchloremic (*n* = 82) or a lactic (*n* = 88) origin.

Patients with hyperchloremic acidosis were women in 32 (39%) cases and men in 50 (61%) cases. Most of patients with lactic acidosis (LA) were men, with 67 (76%) cases compared to 21 (24%) for women (*p* = 0.03). There was no difference in the ages of patients between the two groups.

#### 3.2.1. Pre-Hospital Fluid Resuscitation

We did not find any impacts of NS on the type of acidosis, since patients with hyperchloremic acidosis received NS in 69 (84%) cases and patients with lactic acidosis received NS in 76 (86%) cases, which is not statistically different (*p* = 0.68). Comparing HCA and LA groups, we found a mean pre-hospital fluid volume of 502 (SD 356) mL vs. 455 (SD 312) mL (*p* = 0.35).

#### 3.2.2. Initial Condition on Arrival in the ED

The mean HR in the ED was 92 (SD 21) bpm in the HCA group and 99 (SD 22) bpm in the LA group (*p* = 0.02).

In the ED, patients with LA had a more profound acidemia with a mean pH of 7.25 (SD 0.02) versus 7.31 (SD 0.09) for those with HCA (*p* = 0.003) and a mean BE of −7.4 (SD 5.6) mEq/L versus −5.9 (SD 3.7) mEq/L (*p* = 0.04), respectively.

Median ED LOS was 72 (IQR 73) min for patients with HCA and longer for patients with LA with a median 100 (IQR 260) min (*p* = 0.02).

#### 3.2.3. Evolution and in-Hospital Interventions

There was no significant difference in the rates of ICU admission between the HCA group (38%) and the LA group (49%), nor in the difference in ICU LOS between the two groups.

Regarding total intrahospital fluid volume, there was no difference when comparing the HCA and LA groups, with a median of 1850 (IQR 3500) mL and 2900 (IQR 3835) mL, respectively (*p* = 0.79). Nevertheless, the fluid volume administered in the ICU was 3746 (SD 2907) mL in the HCA group and 2635 (SD 1683) mL in the LA group, which is just statistically different (*p* = 0.04).

An abnormal baseline creatinine level was seen in 20 (27%) of patients with HCA and in 33 (42%) patients with LA (*p* = 0.05). There was no difference between the two groups concerning the development of AKI during the hospital stay. There were no significant differences in times to BE and lactate clearance.

The median hospital length of stay in HCA patients was 7 (IQR 15.3) days vs. 9.6 (IQR 18) days for LA patients (*p* = 0.046).

#### 3.2.4. Mortality

There was also no difference in mortality between the two groups. The 48-h mortality was 4% (*n* = 3) for patients with HCA and 2% (*n* = 2) for patients with LA (*p* = 0.59). The 30-day mortality was 7% (*n* = 6) in both groups.

## 4. Discussion

In the current study, three out of four patients received NS during the pre-hospital phase, and less than one out of ten received BS, a prevalence similar to that found by Naumann et al. in a study from the United Kingdom [18]. This finding may be surprising, because it shows that, despite the recommendation for administration of BS, NS is still widely used in the field. However, there was a trend towards an increased administration of BS and a decrease in NS administration during the second half of the study period (2014–2019). This shift in practice may in fact be due to the introduction of new practice recommendations [19]. It is well known that there is usually a time lag between the diffusion of new knowledge and the introduction and application thereof.

The results of the present study point in the same direction as the latest European guidelines on the management of major bleeding in trauma patients, which, despite the lack of clear evidence, recommend the administration of BS as the initial fluid for trauma patients without an associated traumatic brain injury (TBI) [20].

Balanced solution was predominantly given to patients brought in by helicopter, whereas the administration of normal saline was more evenly distributed for patients brought in by helicopter, and by medicalized and non-medicalized ambulance. Since helicopter transport always implies the presence of more experienced physicians, this may explain why most patients receiving BS were observed in this setting. These patients are usually also more severely injured than patients transported by ambulance, which may in turn explain why patients in the BS group received a mean volume of 763 mL, which was significantly higher than that of the patients who received NS.

Despite the unequal administration of NS and BS among the patients in our study, we did not observe a difference in the incidences of HCA and LA on arrival in the ED. Also, there was no association between the type of fluid administered during the pre-hospital phase and the presence of HCA in the ED. This result may be due to the fact that the mean fluid volumes administered were lower than in studies which demonstrated an association between fluid type and deterioration in the acid-base status [5,21,22,23,24].

The administration of low to moderate volumes of NS therefore appears to be associated with a lower risk, which is in agreement with the latest guidelines, suggesting that no more than 1000 to 1500 mL of NS should initially be administered to trauma patients [20].

In the present study, the predominant origin of metabolic acidosis was hyperchloremic in women, and lactic in men. This may be due to the tendency for men to sustain more severe injuries and therefore a higher incidence of lactic acidosis.

Neither the pre-hospital administration of NS, nor the presence of HCA in the ED was associated with any differences in mean intrahospital fluid volume administered. The only exception was observed in the ICU, where patients with HCA in the ED received significantly more fluid than patients with LA. One possible explanation for this observation might indeed be erroneous interpretations of the metabolic acidosis and therefore inappropriate fluid loading, as described in a prior study [16]. However, despite the higher ICU fluid volume administered to patients with HCA in the ED, neither the type of fluid administered in the pre-hospital setting nor the type of metabolic acidosis had an influence on the need for, or the duration of, invasive ventilation, which is a result similar to that of a randomized trial, the BaSICS study [25].

There also was no difference in ICU length of stay between patients who had HCA or LA. Therefore, even if the higher fluid volume were iatrogenic due to erroneous interpretation of the metabolic acidosis, it had no clinical consequences in the present study.

In contrast to the study by Zampieri et al. [25], patients who received BS had a longer ICU stay and went more frequently to the OR compared to patients in the NS group. This difference could be due to the fact that patients who received BS were almost all transported by helicopter, a mode of transportation mainly used for severely injured patients. This hypothesis is supported by the fact that patients who received BS were intubated more frequently, had lower GCS scores on arrival in the ED, and required more PRBC transfusion during the first 24 h of hospitalization. However, no difference in ISS scores was observed between the NS and the BS groups.

The total length of hospital stay was longer for patients with LA. This is likely because the source of acidosis was actual tissue hypoperfusion, and not “factitious” hypoperfusion due to hyperchloremia. When comparing the types of pre-hospital fluids, hospital lengths of stay did not differ.

In several large studies comparing the administration of NS or BS, the prevalence of AKI consistently emerges as one of the main complications of NS administration. Although a recent study showed an increased risk of AKI when administering NS [23], our study did not show any difference in AKI between fluid types or origins of metabolic acidosis. This is in line with other several recently published studies showing that patients receiving NS or exhibiting an HCA were not at an increased risk of developing AKI [22,24,25,26].

In our cohort, patients with LA more often had abnormal baseline creatinine levels than others, which is probably explained by the tissue hypoperfusion at the origin of the LA. These patients also had a significantly higher HR in the ED than patients with an HCA.

Another widely studied outcome in this area is the impact of fluid type on mortality. Even though hyperchloremia was linked to increased mortality in certain studies [10,11,13,15], the type of fluid administered has never been shown to increase mortality [21,23,24,25,26]. In the present study, no differences in 48-h and 30-day mortalities were found between the types of pre-hospital fluids and the origins of metabolic acidosis.

## 5. Limitations

The current study has two major limitations. First, this is a retrospective study based on the registry of a single hospital center, which limits the generalizability of the results. Second, we know that the documentation of liquids administered in pre-hospital is variable with a frequent lack of reporting [27], a difficulty that we also faced when collecting pre-hospital data as well as laboratory data.

## 6. Conclusions

In summary, the dominantly administered pre-hospital fluid is still NS, except for patients brought in by helicopter. However, there was no association between the type of fluid administered during the pre-hospital phase and the presence of HCA in the ED.

Concerning the administered intrahospital fluid volumes, neither the type of pre-hospital fluid, nor the type of metabolic acidosis in the ED had an influence on the mean intrahospital volume administered. Nevertheless, patients with HCA in the ED received more fluid in the ICU than those with LA, and there were more PRBC transfusions among patients who received BS.

Regarding the duration of total hospital and ICU lengths of stay, they were longer for patients with LA and those who received BS, respectively.

Finally, we found no influence of pre-hospital fluid types or origins of acidosis on the duration of invasive ventilation, the incidence of AKI, and 48-h or 30-day mortalities.

## Figures and Tables

**Table 1:** Study population comparing “NS” group with “Balanced” and “None” group (*n* = 1018).

	All *n* = 1018 (100%)	NS *n* = 778 (76%)	Balanced *n* = 81 (8%)	None *n* = 159 (16%)	*p*
Age (y) mean (SD)	44.4 (20.4)	44.3 (20.8)	44.6 (16.6)	45 (20.5)	0.88
Sex (m/f) *n* (%)					0.06
- Female	248 (24)	177 (23)	21 (26)	50 (31)	
- Male	770 (76)	601 (77)	60 (74)	109 (69)	
Mechanism, *n* (%)					0.05
- Road traffic accident	526 (52)	413 (53)	47 (58)	66 (42)	
- Fall	371 (36)	274 (35)	28 (35)	69 (43)	
- Penetrating	103 (10)	80 (10)	5 (6)	18 (11)	
- Other	18 (2)	11 (1)	1 (1)	6 (4)	
Mode of transportation, *n* (%)					<0.001
- Ambulance	120 (12)	83 (11)	2 (2)	35 (22)	
- Medicalized ambulance	358 (35)	306 (39)	3 (4)	49 (31)	
- Helicopter	540 (53)	389 (50)	76 (94)	75 (47)	
Pre-hospital intubation, *n* (%)	184 (18)	139 (18)	22 (27)	23 (14)	0.041
Pre-hospital fluid volume (mL), mean (SD)	459 (431)	520 (387)	763 (607)	0	<0.001
GCS in ED, mean (%)	12 (4.9)	12 (4.8)	11 (5.2)	12 (4.5)	0.01
Systolic blood pressure in ED, mean (%)	135 (26)	135 (26)	133 (28)	137 (25)	0.97
Heart rate in ED, mean (%)	91 (21)	90 (22)	92 (23)	91 (21)	0.51
Vasopressor use in ED, *n* (%)	219 (22)	166 (21)	25 (31)	28 (18)	0.06
ISS, mean (SD)	16 (12)	16 (12)	19 (14)	14 (11)	0.41
Arterial blood gas (versus venous), *n* (%)	782 (77)	614 (79)	59 (73)	109 (69)	0.01
pH, mean (SD)	7.34 (0.11)	7.34 (0.12)	7.31 (0.11)	7.35 (0.10)	0.16
ED Lactate, mean (SD)	2.78 (2.67)	2.74 (2.61)	2.75 (1.98)	3.12 (3.23)	0.03
ED Lactate, median (IQR)	2.1 (1.9)	2 (1.8)	2.3 (1.7)	2.3 (2)	
ED BE, mean (SD)	−3.6 (4.5)	−3.6 (4.6)	−4.2 (4.6)	−3.4 (4.2)	0.21
ED chloride, mean (SD)	107 (5)	107 (5)	107 (4)	NA	0.87
Peak chloride, mean (SD)	111 (7)	111 (7)	111 (6)	NA	0.90
ED Sodium, mean (SD)	142 (4)	142 (4)	141 (4)	NA	<0.001
ED Strong ion difference, mean (SD)	33 (5)	34 (5)	33 (5)	NA	0.21
ED Creatinine above N, *n* (%)	194 (25)	180 (26)	14 (18)	0 (0)	0.25
Peak creatinine, mean (SD)	97 (43)	97 (42)	95 (45)	NA	0.81
AKI during hospital stay, *n* (%)	41 (5)	34 (5)	7 (10)	0 (0)	0.07
Time period, *n* (%)					<0.001
- 2008–2013	577 (57)	472 (61)	31 (38)	74 (47)	
- 2014–2019	441 (43)	306 (39)	50 (62)	85 (53)	
Acid-Base status in ED, *n* (%)					0.30
- Metabolic acidosis	559 (55)	425 (55)	48 (59)	86 (55)	
- Normal	255 (25)	197 (25)	23 (28)	35 (22)	
- Other	204 (20)	156 (20)	10 (12)	38 (24)	
Intrahospital fluid volumes					
- ED, mean (SD)	658 (661)	668 (650)	717 (612)	579 (723)	0.09
- ED, median (IQR)	500 (750)	500 (700)	500 (700)	500 (250)	
- OR (*n* = 313), mean (SD)	2336 (1780)	2419 (1717)	2015 (1124)	2145 (256)	0.90
- OR, median (IQR)	2000 (2000)	2000 (2000)	2000 (1200)	1500 (1500)	
- ICU (*n* = 359), mean (SD)	2397 (3245)	2381 (3451)	2799 (2666)	2206 (2399)	0.13
- ICU, median (IQR)	2000 (3550)	2000 (3575)	2500 (4000)	1655 (3150)	
- Total, mean (SD)	2221 (3084)	2234 (3193)	2897 (2883)	1813 (2546)	0.08
- Total, median (IQR)	1000 (2700)	2000 (3575)	2000 (3700)	500 (2250)	
Total PRBC 24 h, mean (SD)	0.59 (2.13)	0.53 (1.85)	1.35 (4.08)	0.50 (1.91)	0.005
Total PRBC 24 h, median (IQR)	0 (0)	0 (0)	0 (0)	0 (0)	
Went to OR, *n* (%)	313 (31)	236 (30)	39 (48)	38 (24)	0.001
Went to ICU, *n* (%)	359 (35)	272 (35)	35 (43)	52 (33)	0.26
Invasive ventilation, *n* (%)	314 (31)	240 (31)	30 (37)	44 (28)	0.33
Duration inv. Vent. (d), mean (SD)	4.9 (6.2)	4.7 (5.8)	5.6 (6.7)	5.2 (7.9)	0.87
Duration inv. Vent. (d), median (IQR)	2 (7)	2 (7)	3.5 (7)	2 (7.5)	
Time to normal BE (>−2) (h), mean (SD)	24.5 (77.1)	24.8 (28.1)	22.2 (24.1)	NA	0.53
Time to normal BE, median (IQR)	12 (17.7)	11.9 (19)	14.7 (15.4)	NA	
Time to clearance Lac (<2.44), mean (SD)	12 (22.3)	10.7 (19.5)	20.4 (35.3)	NA	0.37
Time to clearance Lac, median (IQR) (h)	5 (8.9)	5.2 (8.6)	4.4 (11.8)	NA	
ED LOS (min), mean (SD)	112 (177)	105 (162)	131 (212)	139 (224)	<0.001
ED LOS (min), median (IQR)	55 (47)	55 (43)	63 (63)	57 (80)	
ICU LOS (d), mean (SD)	2.4 (5.8)	2.3 (5.3)	3.5 (8.6)	2.2 (5.9)	0.006
ICU LOS (d), median (IQR)	0 (1.5)	0 (1.5)	0 (3.1)	0 (1.4)	
Hospital LOS (d), mean (SD)	11.4 (14.5)	11.1 (13.5)	13.4 (17.2)	11.9 (17.4)	0.15
Hospital LOS (d), median (IQR)	5.9 (15)	5.8 (15.5)	7 (15.5)	5.5 (14)	
Mortality (48 h), *n* (%)	70 (7)	55 (7)	7 (9)	8 (5)	0.53
Mortality (30 days), *n* (%)	113 (11)	87 (11)	10 (12)	16 (10)	0.86

NA = not available.

**Table 2:** Comparing patients with hyperchloremic and lactic acidosis (*n* = 170).

	All (*n* = 170)	Hyper-Cl (*n* = 82)	Lactic (*n* = 88)	*p*
Age (y), mean (SD)	44.3 (19.5)	43.7 (18.4)	44.9 (20.5)	0.71
Sex, *n* (%)		0.03		0.03
- Female	53 (31)	32 (39)	21 (24)	
- Male	117 (69)	50 (61)	67 (76)	
Mechanism, *n* (%)		0.12		0.12
- Road traffic accident	90 (53)	46 (56)	44 (50)	
- Falls	58 (34)	25 (30)	33 (38)	
- Penetrating	18 (11)	11 (13)	7 (8)	
- Other	4 (2)	0	4 (5)	
Pre-hospital intubation, *n* (%)	30 (18)	13 (16)	17 (19)	0.55
Pre-hospital fluid volume (mL), mean (SD)	478 (334)	502 (356)	455 (312)	0.35
Pre-hospital fluid type, *n* (%)		0.68		0.68
- Normal saline	145 (85)	69 (84)	76 (86)	
- Balanced solution	25 (15)	13 (16)	12 (14)	
GCS in ED, mean (SD)	12 (5)	12 (5)	11 (5)	0.08
Systolic blood pressure in ED, mean (SD)	132 (25)	132 (25)	131 (28)	0.75
Heart rate in ED, mean (SD)	95 (21)	92 (21)	99 (22)	0.02
Vasopressor use in ED, *n* (%)	43 (25)	16 (20)	27 (31)	0.09
ISS, mean (SD)	19 (12)	18 (12)	20 (13)	0.30
pH, mean (SD)	7.28 (0.14)	7.31 (0.09)	7.25 (0.02)	0.003
Lactate, mean (SD)	3.53 (3.15)	2.18 (1.17)	4.79 (3.83)	<0.001
ED BE, mean (SD)	−6.7 (4.8)	−5.9 (3.7)	−7.4 (5.6)	0.04
ED chloride, mean (SD)	107 (5)	110 (5)	105 (3)	<0.001
Peak chloride, mean (SD)	111 (8)	113 (8)	110 (7)	0.001
ED sodium, mean (SD)	140 (4)	140 (4)	140 (3)	0.16
ED strong ion difference, mean (SD)	33 (5)	30 (4)	36 (3)	<0.001
ED creatinine above N, *n* (%)	53 (35)	20 (27)	33 (42)	0.05
Peak creatinine, mean (SD)	102 (45)	99 (51)	106 (39)	0.33
AKI during hospital stay, *n* (%)	13 (9)	8 (11)	5 (7)	0.33
Intrahospital fluid volume				
- ED, mean (SD)	616 (519)	546 (425)	681 (589)	0.09
- ED, median (IQR)	500 (500)	500 (350)	500 (600)	
- OR (*n* = 81), mean (SD)	2244 (1530)	2220 (1232)	2269 (1813)	0.87
- OR, median (IQR)	2000 (2000)	2000 (1500)	2000 (2000)	
- ICU (*n* = 74), mean (SD)	3101 (2325)	3746 (2907)	2635 (1683)	0.04
- ICU, median (IQR)	2730 (2700)	3500 (3600)	2500 (1790)	
- Total, mean (SD)	3034 (3032)	3099 (3512)	2974 (2522)	0.79
- Total, median (IQR)	2350 (3800)	1850 (3500)	2900 (3835)	
Total PRBC 24 h, mean (SD)	0.6 (1.6)	0.6 (1.5)	0.7 (1.7)	0.70
Total PRBC 24 h, median (IQR)	0 (0)	0 (0)	0 (0)	
Went to OR, *n* (%)	81 (48)	42 (51)	39 (44)	0.37
Went to ICU, *n* (%)	74 (44)	31 (38)	43 (49)	0.15
Invasive ventilation, *n* (%)	63 (37)	29 (35)	34 (39)	0.66
Duration invasive ventilation, mean (SD)	4 (4.7)	3.7 (4)	4.3 (5.2)	0.59
Duration invasive ventilation, median (IQR)	2 (7)	2 (4)	2 (7)	
Time to normal BE (>−2), mean (SD)	24.3 (29)	25.7 (32.9)	23.1 (25.4)	0.67
Time to normal BE (>−2), median (IQR)	16.8 (27.2))	16.4 (27.6)	16.8 (26.8)	
Time to clearance Lac (<2.44), mean (SD)	10.9 (20.3)	6.1 (5.4)	12.6 (23)	0.30
Time to clearance Lac (h), median (IQR)	3.9 (8.9)	4.2 (8.3)	3.9 (9.1)	
ED LOS (min), mean (SD)	176 (224)	135 (207)	214 (232)	0.02
ED LOS (min), median (IQR)	85 (157)	72 (73)	100 (260)	
ICU LOS (d), mean (SD)	2.4 (4.6)	1.9 (3.6)	2.8 (5.4)	0.22
ICU LOS (d), median (IQR)	0 (2.7)	0 (2.5)	0 (2.9)	
Hospital LOS, mean (SD)	14 (15.4)	11.5 (12.2)	16.2 (17.6)	0.046
Hospital LOS, median (IQR)	8.6 (16)	7 (15.3)	9.6 (18)	
Mortality (48 h), *n* (%)	5 (3)	3 (4)	2 (2)	0.59
Mortality (30 days), *n* (%)	12 (7)	6 (7)	6 (7)	0.90

**Table 3:** Patients with metabolic acidosis by severity (*n* = 559).

	Normal pH (7.35–7.45) Acidosis (*n* = 209)	Mild (pH 7.20–7.34) Acidosis (*n* = 273)	Severe (pH < 7.20) Acidosis (*n* = 77)	*p*
Pre-hospital fluid type, *n* (%)				
- Normal saline	156 (74.6)	208 (76.2)	61 (79.2)	0.72
- Balanced solution	11 (5.3)	29 (10.6)	8 (10.4)	0.10
- None	42 (20)	36 (13.2)	8 (10.4)	0.02
AKI during hospital stay, *n* (%)	7 (3.3)	19 (7)	5 (6.5)	0.27
Mortality (48 h), *n* (%)	2 (1)	22 (8)	26 (33.8)	<0.001
Mortality (30 days), *n* (%)	7 (3.3)	36 (13.2)	35 (45.5)	<0.001

## Data Availability

The data are available from the corresponding author upon request. The data are not publicly available due to institutional regulations, limiting access to data from the trauma registry.
